# Ovarian Follicle Growth during Lactation Determines the Reproductive Performance of Weaned Sows

**DOI:** 10.3390/ani10061012

**Published:** 2020-06-10

**Authors:** Tania P. Lopes, Lorena Padilla, Alfonso Bolarin, Heriberto Rodriguez-Martinez, Jordi Roca

**Affiliations:** 1 Department of Medicine and Animal Surgery, Veterinary Science, University of Murcia, 30100 Murcia, Spain; taniamarisa.piedade@um.es (T.P.L.); lorenaconcepcion.padilla@um.es (L.P.); 2 AIM Iberica, Topigs Norsvin España, 20290 Madrid, Spain; abolarin@aimiberica.com; 3 Department of Biomedical and Clinical Sciences (BKV), Linköping University, SE-58185 Linköping, Sweden; heriberto.rodriguez-martinez@liu.se

**Keywords:** lactation, ovarian follicles, pig, ultrasonography, weaning

## Abstract

**Simple Summary:**

In this field study, the ovaries of weaned (*n* = 191, experiment 1) and lactating (*n* = 40, experiment 2) sows were transrectally scanned to measure the diameter of the follicles. Both the weaned and lactating sows showed great variability in the diameter of the ovarian follicles, indicating that the variability at weaning already existed during early lactation and was carried over to weaning. Sows with small follicles at weaning showed low reproductive performance and were more frequent among those with fewer farrowings and those weaned in summer–autumn.

**Abstract:**

Factors causing variability in ovarian follicle size among weaned sows are not well known. This field study aimed to disclose influencing factors and evaluate if the differences at weaning were established during lactation. Ovaries were scanned using transrectal ultrasound. The first experiment was conducted over a year with 191 randomly chosen sows that were hierarchically grouped (*p <* 0.001) according to ovarian follicle diameter reached at weaning: Small (0.20–0.30 cm; *n* = 37), medium (0.31–0.39 cm; *n* = 75), and large (0.40-1.00 cm; *n* = 69). Sows with small follicles showed a higher incidence of post-weaning anestrus (*p* < 0.01), longer wean-to-estrus/ovulation intervals (*p <* 0.01) and farrowing smaller litters (*p* < 0.05). Ovaries with small follicles were more common among sows weaned in summer–autumn than in winter–spring (*p <* 0.01) and among sows of lower parity (1–3) (*p <* 0.05). In the second experiment, with 40 sows randomly chosen at farrowing, the ovaries were scanned at 7, 14, and 21 d post-partum. Sows showed great variability in ovarian follicular size during lactation with a consistent relationship between the three measurement times (r = 0.84, *p <* 0.01). Follicle size was smaller in sows nursing in summer–autumn than in winter–spring (*p <* 0.05). In conclusion, early lactation dictates the great variability in ovarian follicular diameter at weaning shown by sows. Sows with smaller follicles at weaning had longer intervals for estrus and ovulation and smaller litters at farrowing and they were in greater numbers among sows weaned during the summer and fall and among those with fewer previous farrowing.

## 1. Introduction

Pig production is an economic activity whose profitability relies largely in an efficient reproductive management of sows [1,2]. Achieving such efficiency involves reducing non-productive days, which will allow more piglets to be weaned per sow and year [3]. Sows with one or more farrowings represent around 80% of the total sow population in breeding pig farms. They are inseminated at the first estrus after weaning, occurring most often between the third and fifth day after weaning [4]. Unfortunately, a variable number of weaned sows do not show estrus within this period, which delays their insemination and increases the number of non-productive days [5,6]. Moreover, long weaning-to-estrus intervals are related to increased pregnancy losses, decreased farrowing rates, and smaller litter sizes [5,7].

Noteworthy, sows show marked variability in the size of ovarian follicles at weaning [8,9]. Sows with small follicles at weaning tend to have longer weaning-to-estrus intervals [5,10]. Since the factors causing this variability in ovarian follicle size at weaning are not yet completely exposed [7], their elucidation would help to design effective management strategies to reach and maintain optimal weaning-to-estrus intervals. The seasons of the year, parity, body condition, and lactation duration are considered among the main factors influencing ovarian follicular growth in sows [7]. Revealing the role played by the season of the year would be particularly interesting since seasonal changes in daylight and air temperature are well-known factors influencing the reproductive performance of pigs, including those raised indoors [11,12,13,14]. Consequently, the present study aimed to reveal whether the above-mentioned putative causal factors would explain the differences between sows in follicular size at weaning and to assess whether such differences were already established during lactation. 

## 2. Materials and Methods 

### 2.1. Farm Location, Sows, and Reproductive Management

The experiments were carried out following the European Union guidelines for animal experimentation and were approved by the Bioethics Committee of the University of Murcia (research code: 639/2012). The experiments were conducted in a breeding pig farm of the Region of Murcia, Spain (37°59′ N, 1°08′ W). This geographic area is characterized by daylight varying from 9 h 32 min in the winter solstice to 14 h 48 min in the summer solstice yielding high environmental temperatures. During the experimental period, maximum temperature averaged 33.7 °C in summer, 18.4 °C in winter and averaged 25.6 °C throughout the year.

The commercial farm where the study was carried out held 1850 hybrid (Large White × Landrace) breeding sows. The sows had free access to water and ate a commercial feed switching in composition, namely 13% CP; 6.59% CF and 2900 kcal/kg ME during gestation with a feed intake ranging from 2.3 to 2.8 kg per day. Differently, lactating sows had an average intake of 4.5 kg per day of a commercial feedstuff composed by 17.50% of CP; 4.17% of CF and 3200 kcal/kg ME. The farm did not have a controlled environment outside of farrowing. Farrowing room temperature was adjusted to 24 °C by using evaporative cooling systems and air extractors to achieve welfare comfort levels.

At weaning, sows (up to six farrowings) were allocated into individual crates for estrus detection. Detection started the day after weaning, with two sessions per day, usually at 7:00–8:00 and 18:00–19:00. Detection was performed by experienced farm staff in the presence of a healthy adult boar. The start of the estrus was defined as 6 h before the first time that the sows showed an immobile standing in response to back pressure tests during snout-to-snout contact with the boar located in the alley in front of the crate. Once in estrus, sows were two or three times post-cervically inseminated using liquid semen doses of 40 mL containing 1.5 × 10^9^ total spermatozoa. The semen doses were purchased from an independent boar station (AIM Iberica, Topigs Norsvin España, Spain) and remained stored at 15–17 °C for 12–48 h before use. Twenty-eight days after insemination, sows were grouped in pens of 25 m^2^ (10 sows/pen), where they stayed until seven days before the expected parturition day. Then, sows were placed into individual farrowing crates until weaning. The lactation period length ranged from 20 to 28 d. 

### 2.2. Transrectal Ultrasonography

Functional activity of ovaries was checked by transrectal ultrasonography, a validated tool for measuring and counting physiological and pathological ovarian structures [15]. Transrectal ultrasonography was performed using LOGIQ Book XP ultrasound instrument equipped with a 4–10 MHz multivariable frequency linear transducer (General Electric Co, Solingen, Germany), following the procedure described by Bolarin et al. [16]. Briefly, the transducer, placed in the palm of the hand and ventrally oriented, was inserted into the rectum about 35–45 cm, the expected location of the ovaries. The two ovaries were scanned separately and frames from each ovary were recorded using digital cinema technology. Thereafter, either physiological or pathological functional structures were counted and measured using the calibrated measurement software included in the ultrasound scanner. Spherical anechoic ovarian structures with diameter up to 1.00 cm, showing thin borders and, occasionally, with irregular outlines, were defined as follicles. Similar structures, but with a diameter equal or greater than 1.10 cm, were defined as cysts. Occurrence of ovulation was recorded when the number of follicles was noticeably lower than that in the previous count. Circular, homogeneous and hypo-echoic structures were identified as corpora lutea. The number of follicles was registered only when their diameter reached 0.20 cm. The diameter of three of the largest follicles per ovary was then measured and the arithmetic mean of the diameters of these follicles of both ovaries was defined as the ovarian follicular size. 

### 2.3. Experiments

Experiment 1. Factors influencing ovarian follicle size at weaning and its influence in post-weaning reproductive performance.

A total of 191 sows, 95 in summer–autumn (SA, between July and October) and 96 in winter–spring (WS, between February and May), were randomly selected before weaning, in groups of 20–25 sows. The sows were of parity (P) 1–6, distributed as P1 = 27, P2 = 45, P3 = 24, P4 = 44, P5 = 30, and P6 = 21, and followed a similar distribution among SA and WS (parity 3.22 ± 0.17 and 3.49 ± 0.16, respectively; *p* > 0.05). Individual body condition was scored on a scale of 1 to 5 following the procedure described by Charette et al. [17]. Sows in SA and WS showed similar body condition (3.21 ± 0.39 and 3.25 ± 0.44, respectively; *p* > 0.05). Lactation length was recorded at weaning.

The weaned sows were subjected to estrus detection following the procedure described above, recording the onset and end of estrus. The ovaries were scanned once a day from weaning to the beginning of estrus and twice a day thereafter, until ovulation. Sows not exhibiting estrus during the first 8 d post-weaning were considered as anestrus. The weaning-to-estrus, estrus-to-ovulation, and weaning-to-ovulation intervals were recorded. The farrowing rate and total number of piglets born per litter were recorded. 

Experiment 2. Ovarian follicle growth during lactation and its influence upon post-weaning reproductive performance.

A total of 40 sows were randomly selected at farrowing, 20 in SA and 20 in WS. They showed similar parities (3.39 ± 0.35 and 3.61 ± 0.39 for SA and WS sows, respectively) and body condition (3.05 ± 0.03 and 3.02 ± 0.03 for SA and WS sows, respectively). The ovaries of the sows were transrectally scanned as described above at days 7, 14, and 21 of lactation to measure follicle size.

### 2.4. Statistical Analysis

**Figure 1:**
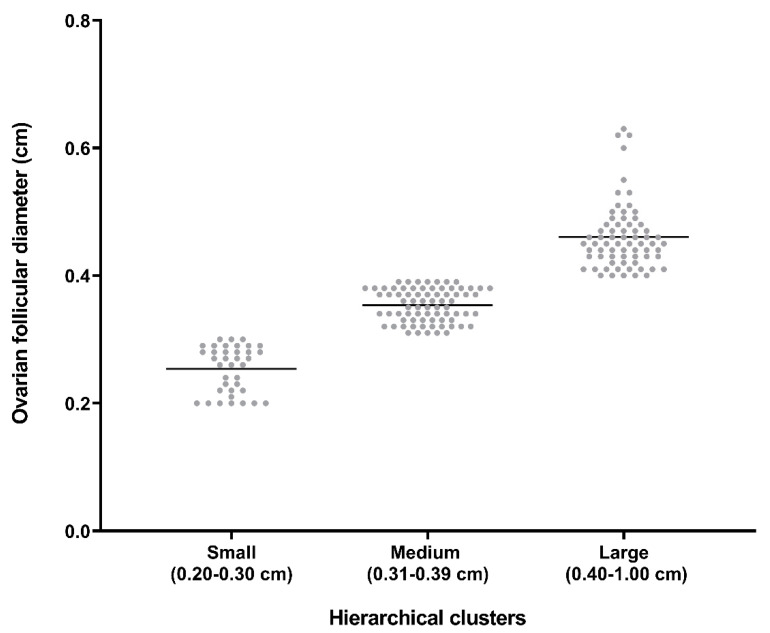
Distribution of the sows in three clusters, defined objectively by hierarchical cluster analysis (*p* < 0.001), according to average ovarian follicular diameter at weaning.

Analyses were performed using IBM SPSS Statistics 24.0 (IBM Spain, Madrid, Spain). Wilk–Shapiro test was used for checking normality of count data, and those not normally distributed were log-transformed. In experiment 1, a hierarchical cluster analysis was used to define how sows were objectively grouped according to their average ovarian follicles size at weaning, and three groups of sows were generated as having small (0.20–0.30 cm), medium (0.31–0.39 cm) and large (0.40–1.00 cm) ovarian follicles (Figure 1). A multivariate ANOVA model was used to evaluate the influence of season of the year, body condition, lactation length, and the number of previous farrowing in the ovarian follicular size at weaning. Chi-square test was used for checking differences in the distribution of sows among the different generated groups. One-way ANOVA with post-hoc Tukey HSD test was used to evaluate the influence of follicular size at weaning on the size and number of follicles at the beginning of estrus, in the length of weaning-to-estrus, estrus-to-ovulation, and weaning-to-ovulation intervals and in the number of piglets born per litter. In experiment 2, one-way ANOVA was used to evaluate the influence of seasons of the year (SA vs. WS) in the ovarian follicle size. Data were showed as mean ± SEM (in Tables) and as median together with 5th, 25th, 75th, and 95th percentiles (in Figures). Differences were considered significant at *p* < 0.05 level.

## 3. Results

### 3.1. Experiment 1

Ten of the initial 191 sows (10.53%), all in the WS season, did not show ovarian follicles at weaning. The ovaries of one of these ten sows had follicular cysts and the other 9 had corpora lutea. These 10 sows were not considered in the study, reducing the number of sows to 181, namely 85 in WS and 96 in SA. Considering the clusters of sows defined according to the mean size of the ovarian follicles at weaning, 37 of the 181 sows had small (20.44%), 75 medium (41.44%), and 69 large (38.11%) follicles.

Season of the year and parity influenced (*p* < 0.01) follicular size at weaning (Table 1), but neither body condition (between 2.5 and 3.5) nor lactation length (between 20 and 28 d) influenced follicle size (Table 2). The interaction between season of the year and parity was not significant. There were more sows with small follicles at weaning in SA than in WS, while there were more sows with large follicles in WS than in SA (Table 1). The percentage of sows with small follicles was higher among those of lower parities (1–3) than among those of parity 4–6, while the percentage of sows with large follicles was higher among those of parity 4–6 (Table 1).

Post-weaning, 26 of the 181 sows (14.36%) did not show estrus signs within 8 d and were considered anestrus. These sows started showing estrus signs between 9 and 33 days post-weaning (15.50 ± 1.58 d). Considering the groups of sows defined by their mean follicular size at weaning, the incidence of anestrus was higher (*p* < 0.01) in sows with small follicles (11/37; 29.73%) than among those with medium (10/75; 13.33%) or large follicles (5/69; 7.25%). Of the 26 anestrus sows, more (24) were noted in SA than in WE (2) (*p* < 0.001). 

**Figure 2:**
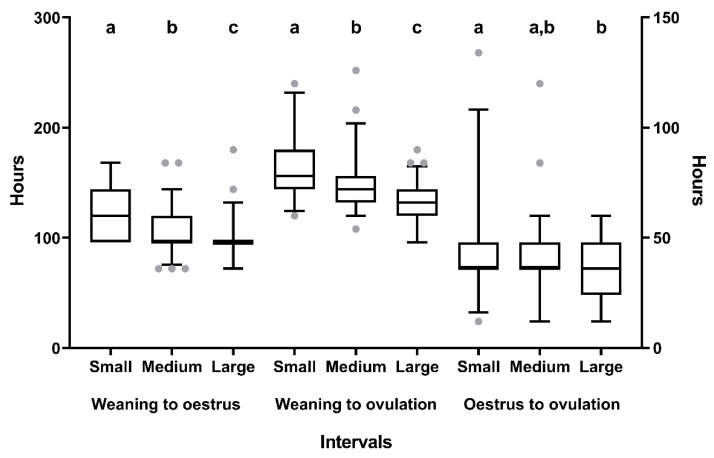
Box–whisker plots showing differences in the intervals between weaning and onset of estrus (left Y axis), weaning and ovulation (left Y axis) and the start of estrus and ovulation (right Y axis) among sows hierarchically grouped according to the mean ovarian follicle diameter at weaning: Small (0.20–0.30 cm), medium (0.31–0.39 cm), or large (0.40–1.00 cm). Boxes enclose the 25th and 75th percentiles; the line is the median; and the whiskers extend to the 5th and 95th percentiles. Grey dots show outliers. a, b, and c indicate significant differences (*p* < 0.05).

For sows oestrous within 8 d after weaning (*n* = 155), follicular size at weaning influenced (*p* < 0.01) the intervals of weaning-to-estrus, estrus-to-ovulation, and weaning-to-ovulation (Figure 2).

Sows with small follicles at weaning showed the longest intervals. The sows with small follicles at weaning also showed both smaller follicles and a tendency to have a lower number of follicles at the beginning of estrus than those with medium and large follicles at weaning (Table 3). 

**Figure 3:**
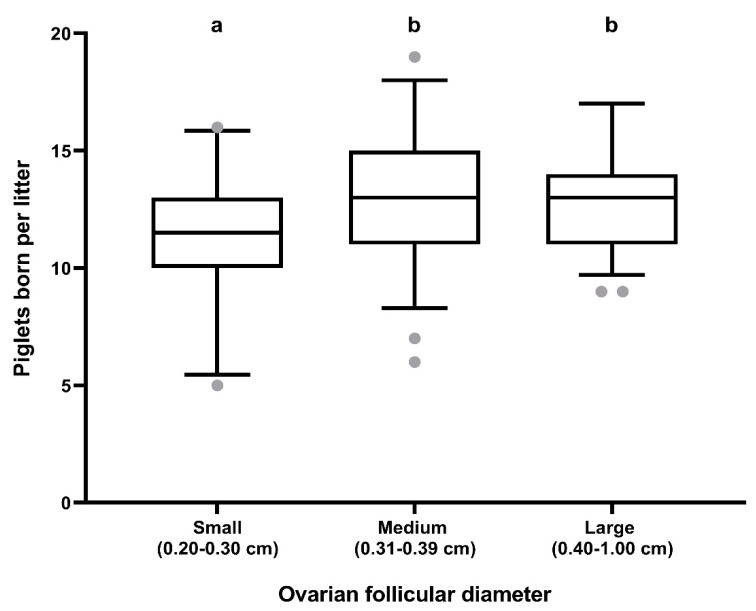
Box–whisker plots showing differences in the number of piglets born per litter among sows hierarchically grouped according to the mean ovarian follicle diameter at weaning: Small (0.20–0.30 cm), medium (0.31–0.39 cm), or large (0.40–1.00 cm) ovarian follicles at weaning. Boxes enclose the 25th and 75th percentiles; the line is the median; and the whiskers extend to the 5th and 95th percentiles. Grey dots show outliers. a and b indicate significant differences (*p* < 0.05).

Follicle size at weaning did not influence farrowing rates (81.93%; 127/155) but it did influence litter size (*p* < 0.05). Sows with small follicles at weaning showed smaller litter sizes that those with medium and large follicles (Figure 3).

### 3.2. Experiment 2

**Figure 4:**
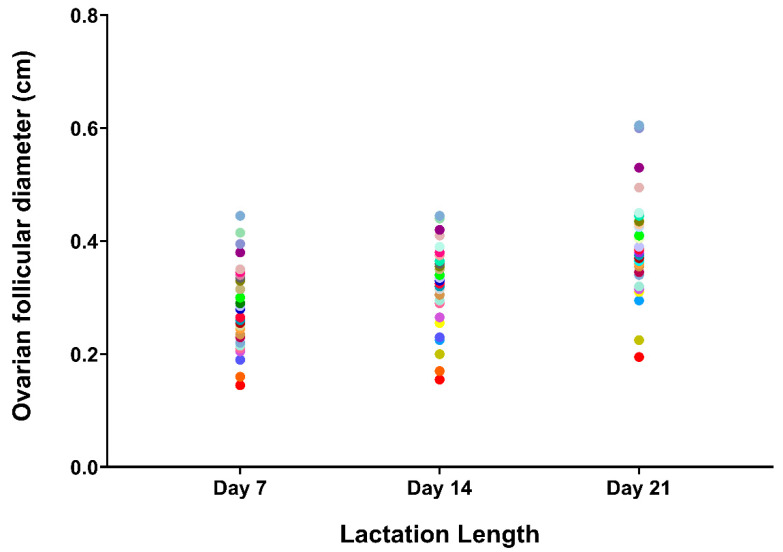
Mean diameter of ovarian follicles in nursing sows (*n* = 40) measured three times throughout the lactation period. Sows are shown with the same color point in the three measurement times.

**Figure 5:**
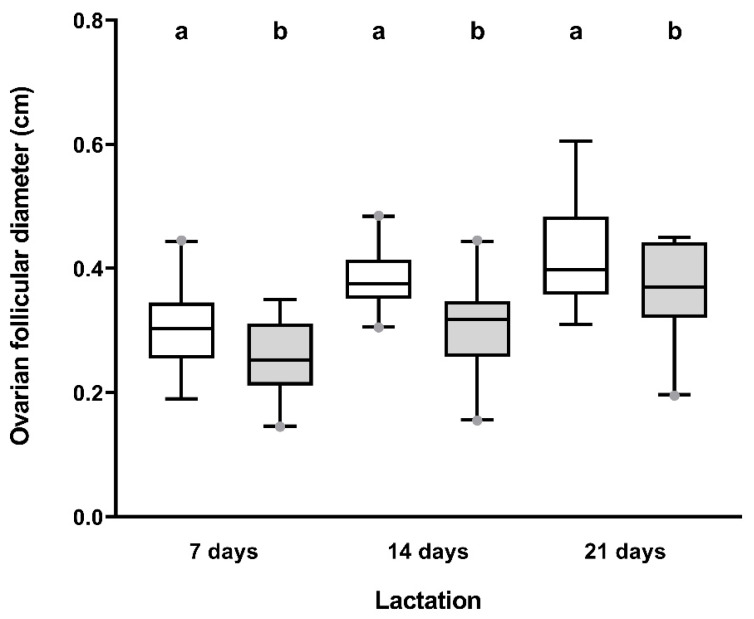
Box–whisker plots showing the ovarian follicular diameter during lactation in sows nursing during winter–spring (white boxes) or summer–autumn (grey boxes). Boxes enclose the 25th and 75th percentiles; the line is the median; and the whiskers extend to the 5th and 95th percentiles. Grey dots show outliers. a and b indicate differences (*p* < 0.05) between season in each measurement days.

The mean follicle size showed great variability between sows at each of the three measurement times (Figure 4), but with a consistent relationship between the three measurements (R^2^ = 0.847). The mean size of follicles in the three measurement days was larger (*p* ≤ 0.05) in the nursing sows of WS than in those of SA (Figure 5).

## 4. Discussion

The present study showed that the size of the ovarian follicles at weaning clearly influenced the post-weaning reproductive performance of the sows and that differences between sows in the size of the follicles at weaning already existed at 7 d after farrowing. These differences were maintained throughout lactation suggesting that follicular measurements during lactation may have utility identifying potentially relatively less fertile sows after weaning. 

The study included two experiments; the first one was focused on factors influencing ovarian follicular size at weaning. The first finding was that a significant number of sows under study had corpora lutea instead of follicles at weaning, which agrees with previous reports [5,18]. These sows probably had ovulated during lactation. During the lactation period, the presence of piglets, the lactation reflex, and the catabolic state derived from milk production inhibit the release of hypothalamic GnRH and, sequentially, the pituitary secretion of LH and FSH, preventing ovarian follicles to grow above 0.50–0.60 cm in diameter [19]. If any of these inhibitory factors is suppressed, follicles could continue to grow and can even ovulate, which usually occurs in some nursing sows [5]. All the sows with corpora lutea at weaning were registered during the WS period. The more comfortable environmental conditions in WS, characterized by milder air temperatures and less daylight, promotes satisfactory feed intake during lactation and thus the possible reversion or attenuation of some the aforementioned inhibitory factors [20]. Ovulation during the lactation period is an important reason to explain the delay in onset of estrus experienced by some weaned sows [21].

Differences in follicular size at weaning were directly related to the post-weaning reproductive potential, which agrees with previous observations [22,23]. The reasons explaining this relationship are not totally clear. However, it is known that a deceleration in the sequence of ovarian events at weaning, specifically regarding follicular growth, delays the onset of estrus after weaning [6,10]. Moreover, follicle size at weaning is related to its size at ovulation [5]. This fact affects the quality of the ovulated oocytes, fertilization rates, and eventually the development of the embryos, as has been evidenced in cows [24]. Furthermore, the corpora lutea resulting from ovulated small follicles would secrete less progesterone, compromising uterine function, and hence, embryo development [11], which can explain the lower litter size in sows with small follicles at weaning. 

The causative factors evaluated in this study were seasons of the year, parity, body condition, and lactation length. Whereas the seasons of the year and the number of parities influenced follicle size at weaning, body condition, and lactation length did not. The four seasons of the year that prevail in temperate regions as summer, autumn, winter, and spring, were grouped according to variations in daylength and accompanying air temperatures, namely of decreasing, towards autumn (SA), and of increasing, towards spring (WS). The influence of air temperature is particularly relevant in the geographical latitude of the farm, since it is high during the summer and early autumn, above the threshold of heat stress on many summer days [25]. The number of sows with small ovarian follicles at weaning was higher in SA than in WS, which was in agreement with previous studies [5,10]. A reversible seasonal endocrine disturbance of ovarian functionality could be the primary cause of the slower growth rate of ovarian follicles in many weaned sows during SA [7,9]. Although not measured in the present study, such endocrine disturbance is presumably caused by a decrease in feed intake during lactation in SA caused by its usual high environmental temperatures [26]. In this regard, disorders on ovarian follicular growth have been related with environmental heat stress [7,27]. Sows weaned during SA showed poorer reproductive performance than those weaned in WS, which was in agreement with previous studies [23,27,28,29,30,31]. The higher incidence of sows with small ovarian follicles at weaning would be the main cause of the comparatively lower reproductive performance showed by the sows weaned in SA. Certainly, sows with smaller ovarian follicles at weaning showed a higher incidence of anestrus, longer weaning-to-estrus or estrus-to-ovulation intervals, and smaller litters born. Then, high ambient temperature in summer and early autumn rather than the relative shortening in daylength during autumn, would be the underlying seasonal cause of the impairment in ovarian follicular growth and the subsequent reproductive disorders experienced by the weaned sows in SA. 

In addition to the seasons of the year, the results also revealed the influence of parity on the follicles size at weaning. The sows were grouped into two groups according to parity number, from one to three and from four to six litters. Those with lower parity had smaller ovarian follicles at weaning than those with higher parity. It is well-known that gilts and primiparous sows have more limited capacity for feed conversion and are more likely to be catabolic at lactation [20,32]. Moreover, sows continue with both limited intake capacity and high energy demand until they reach full body weight, which usually occurs after the third pregnancy. Sows after four litters do not depend so much on these extra requirements [33]. In addition, sows of fourth to sixth litter generally show the best reproductive performance, specifically reflected in litter size and nursing capacity [26].

In contrast with the aforementioned influencing factors, subjectively scored body condition and lactation length did not influence the average size of ovarian follicles at weaning. A good body condition is essential for optimal productive performance of sows [34,35]. However, the predictive value of body condition at weaning on the subsequent reproductive performance of weaned sows is controversial [36,37]. It is clear that a poor body condition at weaning (≤2 of subjective score) negatively influences reproductive performance, by extending weaning-to-estrus interval and decreasing litter sizes [9,36]. However, body condition would have little or no predictive value when the scores were in the range between 2.5 to 3.5 [9], which includes all sows in the present study. The duration of the lactation period was also no related to the ovarian follicular size at weaning. Alike body condition, the short range in the length of lactation shown by the sows under study (between 20 and 28 days), could probably be the reason for the absence of relationship. Similarly, Koketsu [38] in a study performed in Japanese farms showed that lactation duration in the range between 15 and 29 d did not influence the subsequent reproductive performance of the weaned sows.

The observed differences among sows in follicular size at weaning could have been established during lactation. With this hypothesis in mind, our second experiment focused on the evaluation of ovarian follicular growth during lactation and the results revealed clear differences in follicular size among sows from early lactation, at seven days post-partum, which persisted throughout the lactation time. Thus, the sows with the smallest ovarian follicles after farrowing had the smallest follicles during the entire lactation period and at weaning. It seems that the causes of the small ovarian follicles at weaning would begin at post-partum or early lactation. The metabolic rate during lactation differs between sows with equitable feeding [39] and sows with higher weight loss during lactation have longer weaning-to-estrus intervals [40]. The ovarian response to gonadotropins post-partum and during lactation are regulated by insulin and the insulin-like factor (IGF-I), and sows metabolically compromised after farrowing have reduced both insulin and IGF-1 levels [41], which has a negative impact on the ovarian follicular development [19,42,43]. Lucy [41] indicated that this mismatch could persist during lactation and weaning. Our results would confirm this claim by showing that sows with small ovarian follicles at the beginning of lactation period also have small follicles at weaning, causing a delay in the interval between weaning and estrus.

## 5. Conclusions

The results of the present study showed that sows had clear differences in the diameter of the ovarian follicles at weaning and that those with smaller follicles had seriously compromised their following reproductive performance. The results also show that the proportion of sows showing small ovarian follicles is larger during summer–autumn period and in those with less parities. Finally, the results also demonstrated that the differences in follicular diameter on weaning were already established at early lactation. Consequently, the measures to minimize the prevalence of sows with small ovarian follicles at weaning and, thereby, for improving the overall reproductive performance of weaned sows, should be directed to the beginning of lactation period. This should be assessed in further research.

## Figures and Tables

**Table 1:** Distribution of sows (number and proportion in brackets) showing small (0.20–0.30 cm), medium (0.31–0.39 cm) and large (0.40–1.00 cm) ovarian follicles at weaning between the seasons of the year and the number of previous litters. Data are shown as number and proportion (in brackets).

Variable		Ovarian Follicle Diameter at Weaning			Total Sows
		Small	Medium	Large	
Season of the year	Winter–spring	9 (10.59) ^A^	37 (43.53)	39 (45.88) ^a^	85
	Summer–autumn	28 (29.17) ^B^	38 (39.58)	30 (31.25) ^b^	96
Previous litters	1–3	22 (59.46) ^a^	36 (48.00)	20 (28.99) ^A^	78
	4–6	15 (40.54) ^b^	39 (52.00)	49 (71.01) ^B^	103
Total sows		37	75	69	

Within column, A vs. B: *p* < 0.01; a vs. b: *p* < 0.05.

**Table 2:** Body condition and lactation length (mean ± SEM) at weaning in the sows showing small (0.20–0.30 cm), medium (0.31–0.39 cm), and large (0.40–1.00 cm) ovarian follicular diameter at weaning.

Variable	Ovarian Follicle Diameter at Weaning			*p* Value
	Small	Medium	Large	
Body condition (1–5)	3.14 ± 0.05	3.16 ± 0.04	3.22 ± 0.03	0.297
Lactation length (d)	23.08 ± 0.36	23.79 ± 0.32	23.51 ± 0.28	0.575

**Table 3:** Diameter and number (mean ± SEM) of ovarian follicles at onset of estrus in sows with small (0.20–0.30 cm), medium (0.31–0.39 cm), or large (0.40–1.00 cm) ovarian follicular diameter at weaning.

Variable	Ovarian Follicle Diameter at Weaning (cm)			*p* Value
	Small	Medium	Large	
Follicle diameter (cm)	0.68 ± 0.01 ^a^	0.72 ± 0.01 ^b^	0.75 ± 0.01 ^b^	<0.001
Number of follicles ^1^	12.90 ± 2.01	13.71 ± 2.20	13.92 ± 1.73	0.089

^1^ In each ovary.
